# Association of Rapid Early Weight Loss with One-Year Hepatic Steatosis Improvement After Sleeve Gastrectomy: A Retrospective Cohort Study

**DOI:** 10.3390/jcm14207284

**Published:** 2025-10-15

**Authors:** Min Kyoung Jang, Si Yeol Yoon, Jin A An, Ji Soo Kim, Min-Seon Kim, Jung Ah Lee, Chang Seok Ko, Se Hee Min

**Affiliations:** 1 Division of Endocrinology and Metabolism, Department of Internal Medicine, Asan Medical Center, University of Ulsan College of Medicine, Seoul 05505, Republic of Korea; minkyoung.jang.93@gmail.com (M.K.J.); jina0567@naver.com (J.A.A.); mskim@amc.seoul.kr (M.-S.K.); 2 University of Ulsan College of Medicine, Seoul 05505, Republic of Korea; yoonsiyeol1201@gmail.com; 3 Department of Biomedical Science, Asan Medical Institute of Convergence Science and Technology, Seoul 05505, Republic of Korea; gjisu582@gmail.com; 4 Department of Family Medicine, Asan Medical Center, University of Ulsan College of Medicine, Seoul 05505, Republic of Korea; lijunga00@naver.com; 5 Department of Stomach Surgery, Asan Medical Center, University of Ulsan College of Medicine, Seoul 05505, Republic of Korea

**Keywords:** sleeve gastrectomy, early weight loss, hepatic steatosis index, type 2 diabetes mellitus, risk stratification

## Abstract

**Background:** Metabolic dysfunction-associated steatotic liver disease (MASLD), previously referred to as NAFLD, affects nearly one-third of the global adult population and is a leading cause of chronic liver disease, particularly among individuals with obesity and type 2 diabetes (T2DM). Bariatric surgery, including sleeve gastrectomy (SG), has demonstrated a favorable impact on liver fat reduction. However, the predictive value of early postoperative weight-loss trajectories for long-term hepatic improvement remains uncertain, especially in Asian populations, in which MASLD remains understudied despite its increasing prevalence. **Methods:** We retrospectively reviewed 198 adults who underwent SG at a tertiary Korean center between January 2019 and April 2024. After excluding 21 who had postoperative complications or missed early follow-up, 177 patients (mean age 37 ± 9 years; 41% male; mean body mass index (BMI) 40 kg/m^2^) were included in the final analysis. The two-week total weight-loss index (TWL_2W) was calculated, and its association with one-year hepatic steatosis resolution, defined as the normalization of the hepatic steatosis index (HSI < 30) at one year, was assessed using receiver operating characteristic (ROC) analysis to explore the trend toward predictive value. Optimal cut-offs were derived using the Youden Index. Multivariable logistic regression models were adjusted for age, sex, baseline hemoglobin A1c (HbA1c), and BMI. Subgroup analyses were performed according to baseline HSI (35–44, 45–54, ≥55) and type 2 diabetes mellitus (T2DM) status. **Results:** The mean TWL_2W was 7.9 ± 6.6%. A loss of 7.9% optimally predicted HSI values < 30 at one year (area under the curve [AUC] 0.602; unadjusted odds ratio [OR] 2.34; 95% confidence interval [CI] 1.16–4.73). Predictive accuracy improved in T2DM patients (AUC 0.737, 95% CI 0.54–0.95), in whom TWL_2W ≥ 9.1% conferred an adjusted OR 9.12 (95% CI 1.39–59.82), whereas no association was observed in non-diabetic subjects. Stratified analysis showed a pronounced effect in moderate baseline steatosis (HSI 45–54; OR 3.56), but absolute normalization was rare when the baseline HSI was ≥55. Early weight loss was not significantly linked to one-year HbA1c or triglyceride targets. **Conclusions:** An 8–9% reduction in body weight within two weeks of SG was independently associated with the resolution of hepatic steatosis at one year, particularly among patients with T2DM or moderate baseline hepatic steatosis. This simple metric may assist in early risk stratification and guide personalized postoperative care.

## 1. Introduction

The prevalence of obesity has risen steeply over the past three decades. A pooled analysis of 3663 population-based studies showed that in 2022, more than one billion people—over 12% of the world’s population—were living with obesity [1]. A separate global consortium report, covering adults, adolescents, and children across 200 countries, confirmed that this rise has occurred consistently across all age groups and geographic regions from 1990 to 2022 [1]. This global trend has fueled a parallel increase in obesity-related comorbidities. In particular, large cohort studies have demonstrated that obesity and diabetes exert additive effects on atherogenic biomarkers and cardiovascular mortality, with dyslipidemia patterns worsening progressively as body mass index (BMI) increases [2,3].

Among obesity-related comorbidities, metabolic dysfunction-associated steatotic liver disease (MASLD), formerly referred to as non-alcoholic fatty liver disease (NAFLD), has emerged as a major public health concern. The global prevalence of MASLD now exceeds 30% among adults and continues to rise alongside obesity rates [4]. It is currently the leading cause of chronic liver disease and is strongly linked to hepatocellular carcinoma, cardiovascular disease, and type 2 diabetes mellitus (T2DM) [5,6,7]. The prevalence of MASLD is notably high in individuals with obesity or T2DM, affecting approximately 50–90% and 47–63%, respectively [8]. Notably, NAFLD is present in over 90% of individuals with severe obesity who are evaluated for bariatric surgery [9].

Given this substantial disease burden, metabolic–bariatric surgery (MBS) has gained widespread acceptance as an effective treatment modality for patients with obesity and obesity-related metabolic diseases. Current guidelines recommend MBS for adults with BMI ≥ 35 kg/m^2^ regardless of comorbidity status and support its consideration in those with BMI 30–34.9 kg/m^2^ who do not achieve adequate weight loss or metabolic control through nonsurgical means. In recognition of ethnicity-specific risk thresholds, in Asian populations, clinical obesity is recognized at BMI > 27.5 kg/m^2^ [10]. For adults with T2DM, the American Diabetes Association further supports the use of MBS as a weight and glycemic management strategy starting from BMI ≥ 30 kg/m^2^ (or ≥27.5 kg/m^2^ in Asian individuals) in appropriate surgical candidates [11].

Among the available surgical options, sleeve gastrectomy (SG) has become the most widely performed procedure worldwide, now accounting for up to 60% of operations recorded in the eighth International Federation for the Surgery of Obesity and Metabolic Disorders (IFSO) Registry, surpassing Roux-en-Y gastric bypass (RYGB) in all geographic regions [12]. A recent randomized trial showed broadly equivalent excess BMI loss between SG and RYGB at 5 years, though RYGB confers moderately greater total weight loss and dyslipidemia resolution at the cost of higher minor complication rates [13].

Given the widespread adoption of SG and the variability in long-term outcomes, researchers have increasingly investigated whether the magnitude of early postoperative weight loss can serve as a predictor of long-term success following MBS. For example, one large multicenter cohort demonstrated that total weight loss at 3 months strongly correlated with weight trajectory up to 5 years [14]. Conversely, a European multicenter study reported marked inter-individual variability and found that patients with the greatest early weight loss, especially those with the highest weight loss velocity during the 3–6 month period, achieved the most favorable long-term outcomes, highlighting procedure- and population-specific differences [15]. However, these studies vary in design: many include multiple surgical procedures, emphasize anthropometric rather than metabolic endpoints, and apply inconsistent definitions of “early” ranging from preoperative dieting to six-month time points.

In parallel with research on weight trajectories, a number of studies have examined predictors of MASLD remission after bariatric surgery. Key factors associated with hepatic improvement include baseline glycemic status, hepatic histology, improvements in insulin resistance, and early postoperative weight loss [16,17]. Although these findings collectively underscore the metabolic benefits of weight reduction, most outcome assessments are conducted no earlier than 6 to 12 months after surgery, and few have investigated very early predictors during the initial postoperative weeks.

Despite the recognized importance of early postoperative changes, clinical guidance on how much weight loss in the immediate period should prompt additional evaluation or intervention remains vague. While international societies mandate structured follow-up after MBS, they do not define how much weight reduction in the immediate postoperative window should trigger additional intervention [10]. Proof-of-concept data suggest that physiological signals captured within two weeks, such as continuous glucose monitor metrics, can anticipate one-year diabetes remission [18], and meta-regression shows a linear relationship between weight loss and glycated hemoglobin (HbA1c) improvement across interventions [19]. However, no clinically actionable cut-off for early postoperative weight loss has been established.

To address this gap, we hypothesized that the percentage of total weight loss achieved within the first two postoperative weeks independently predicts one-year metabolic outcomes among adults undergoing SG, with a primary focus on hepatic steatosis improvement and secondary outcomes including glycemic control and atherogenic lipid profile. Leveraging a single-center retrospective cohort of 177 SG patients with complete two-week and one-year follow-up data, we aimed to quantify the association between very early weight loss and clinically relevant outcomes, identify optimal early-weight-loss thresholds using receiver operating characteristic (ROC)-based approaches, and explore potential effect modification by baseline hepatic steatosis severity and diabetes status. We propose that identifying a simple, early predictive marker could inform individualized postoperative surveillance and timely adjunctive intervention.

## 2. Materials and Methods

### 2.1. Study Design and Population

This single-center retrospective cohort study was conducted at Asan Medical Center in Seoul, Republic of Korea. Patients who underwent laparoscopic sleeve gastrectomy (LSG) between 1 January 2019, and 30 April 2024, were retrospectively analyzed using data from the Asan Bariatric and Metabolic Surgery Registry. Participants eligible for surgery met at least one of the following criteria: (i) BMI ≥ 35 kg/m^2^ (class II obesity or greater); (ii) BMI ≥ 30 kg/m^2^ with at least one obesity-related comorbidity (e.g., hypertension, T2DM); and (iii) BMI ≥ 27.5 kg/m^2^ with uncontrolled type 2 diabetes mellitus despite appropriate pharmacologic and lifestyle interventions. As part of the surgical eligibility screening, patients with known chronic liver conditions other than MASLD, such as viral hepatitis, autoimmune hepatitis, alcoholic liver disease, or drug-induced liver injury, were not considered surgical candidates and were excluded during the initial preoperative evaluation. For the purposes of this study, patients were further excluded if they did not attend scheduled postoperative follow-up visits, had missing laboratory or anthropometric data, or were prescribed anti-obesity medications during the follow-up period.

This study was conducted in accordance with the Declaration of Helsinki and was approved by the Institutional Review Board of Asan Medical Center (IRB No. S2022-1613-0001). All patient identifiers were anonymized, and data were stored in a secure, password-protected system with restricted access.

### 2.2. Perioperative Management

All patients underwent preoperative evaluation, including routine laboratory tests, esophagogastroduodenoscopy, abdominal computed tomography (CT), and bioelectrical impedance analysis to identify any structural or functional contraindications to surgery and to assess obesity severity and body composition. A psychiatrist also conducted behavioral and psychosocial assessments during outpatient visits, focusing on factors associated with obesity. This evaluation included an assessment of the patient’s perception of their body weight, history of previous weight-loss attempts, and sleep-related symptoms, such as suspected obstructive sleep apnea or poor sleep quality.

Surgical eligibility was determined through a multidisciplinary evaluation involving family medicine, endocrinology, and gastrointestinal surgery specialists. The multidisciplinary team confirmed surgical eligibility through a consensus-based case discussion.

The LSG procedures were performed by a consistent surgical team, with experience of >200 annual gastrectomy cases per surgeon, to ensure standardization and minimize inter-operator variability. The surgical procedure was initiated by using energy devices to carefully divide the branches of the gastroepiploic vessels near the gastric wall along the greater curvature, extending from the antrum to the angle of His. The posterior fundus was fully mobilized by dissecting adhesions within the lesser sac and anterior to the pancreas. The left diaphragmatic crus was exposed to evaluate the presence of a hiatal hernia. Mobilization of the adipose tissue surrounding the gastroesophageal junction allowed for better visualization and safe placement of the staple line. A 36-Fr bougie was inserted trans-orally and positioned along the lesser curvature to guide stapling, which began 4–6 cm proximal to the pylorus. The mobility of the bougie was confirmed by the anesthesiologist before the final stapling, which was performed approximately 1 cm from the angle of His. The gastric specimen was then removed, and the staple line was reinforced using over-sewing techniques to reduce the risk of postoperative bleeding and leakage.

A standardized clinical pathway was implemented for postoperative inpatient care. The patients typically resumed a liquid diet on postoperative day 2 and were discharged without complications on postoperative day 5, following standardized institutional protocols. Routine outpatient follow-up was conducted at 2 weeks and subsequently at 3, 6, 9, 12, 18, and 24 months to monitor postoperative recovery and long-term outcomes. At each visit, anthropometric measurements and laboratory tests were conducted to assess metabolic and weight-related outcomes. The use of anti-obesity medications was not routinely recommended postoperatively, and patients who initiated such medications during the follow-up period were excluded from the final analysis.

### 2.3. Data Collection and Variables

Patient data were retrospectively extracted from the Asan Bariatric and Metabolic Surgery Registry. Baseline demographic and clinical characteristics included age, sex, height, body weight, and BMI, which was calculated as weight in kilograms divided by height in meters squared. Body weight and height were measured using calibrated digital scales (accuracy ± 0.1 kg) and stadiometers (accuracy ± 0.1 cm), respectively.

Comorbidities were identified based on physician diagnosis, laboratory findings, medication used, and imaging results. Four key comorbidities were predefined for this study and used to calculate the number of comorbid conditions per patient:

1. Hypertension, defined as systolic blood pressure ≥ 140 mmHg or diastolic blood pressure ≥ 90 mmHg, or the use of antihypertensive medication [20].
2. Dyslipidemia, defined as total cholesterol (TC) ≥ 240 mg/dL, triglycerides (TG) ≥ 200 mg/dL, low density lipoprotein cholesterol (LDL-C) ≥ 160 mg/dL, or current use of statins or ezetimibe. For patients with diabetes, LDL-C ≥ 100 mg/dL was considered sufficient for the diagnosis [21,22].
3. T2DM, defined by a documented diagnosis, HbA1c ≥ 6.5%, or the use of glucose-lowering medications [23].
4. Fatty liver disease, defined by radiologic evidence of hepatic steatosis on abdominal ultrasound or CT [24,25].

All measurements were performed at standardized time points: baseline (within 2 months prior to surgery) and 2 weeks (±3 days), 3, 6 months, and 1 year (±2 weeks) postoperatively. At each time point, clinical assessments, anthropometric measurements and laboratory tests were conducted according to our institutional follow-up protocol.

The laboratory data included fasting plasma glucose, HbA1c, fasting C-peptide, aspartate aminotransferase (AST), alanine aminotransferase (ALT), TC, LDL-C, high-density lipoprotein cholesterol (HDL-C), TG, serum creatinine, and the estimated glomerular filtration rate (eGFR), calculated using the chronic kidney disease epidemiology collaboration equation. All laboratory measurements were performed after a 12 h overnight fast at the laboratory of Asan Medical Center, following standardized hospital protocols and routine quality control procedures, using a Cobas 8000 modular analyzer (Roche Diagnostics International Ltd., Rotkreuz, Switzerland).

The hepatic steatosis index (HSI) was calculated using the following formula [26]:

HSI = 8 × (ALT/AST) + BMI (+2 if female; +2 if diabetes present)

Early postoperative weight loss was assessed at 2 weeks, and the percentage of total weight loss (%TWL_2W) was calculated as follows:

%TWL_2W = [(preoperative weight − weight at 2 weeks)/preoperative weight] × 100

The Fibrosis-4 (FIB-4) index was calculated as an estimate of liver fibrosis using the following formula [27]:

FIB-4 = (age × AST)/(platelet count × √ALT)

To ensure data accuracy and reliability, all extracted variables were independently reviewed by two researchers. Discrepancies were resolved by consensus, and data entry was cross-checked using the original electronic medical records.

### 2.4. Outcomes

The primary outcome was hepatic steatosis improvement, defined as an HSI < 30 at one-year after surgery, a definition that has been used in previous studies to indicate the absence of MASLD [26,28,29]. Secondary outcomes included the attainment of the following metabolic targets at one-year:

- HbA1c < 6.5% in patients with diabetes or <5.7% in those without diabetes [23];
- LDL-C < 100 mg/dL [22,30];
- TG < 150 mg/dL [22].

Each outcome was coded as a binary variable and used in logistic regression analyses. In addition, a ≥ 35% relative reduction in the HSI was evaluated in an exploratory analysis. This threshold was not a predefined or established cut-off but approximated the mean relative reduction observed in our cohort, and it was analyzed to provide additional insight into the degree of steatosis improvement.

### 2.5. Statistical Analysis

Continuous variables were expressed as means ± standard deviations and compared between groups using independent-samples *t*-tests (or Mann–Whitney U tests for non-normally distributed variables). Categorical variables were presented as numbers (percentages) and compared using Pearson’s χ^2^ tests or Fisher’s exact tests, as appropriate.

First, ROC curves were constructed to evaluate the predictive performance of %TWL_2W for each outcome; areas under the curve (AUCs) were reported. Optimal thresholds for %TWL_2W were identified using the Youden Index and rounded to one decimal place to avoid spurious precision; this threshold was treated as an exploratory, data-driven value rather than an established clinical criterion.

Next, based on this threshold, we conducted a cut-off–based analysis and logistic regression to assess the association between early postoperative weight loss and the achievement of one-year metabolic outcomes, reporting unadjusted and adjusted odds ratios (ORs) with 95% confidence intervals (CIs). Variables with *p* < 0.10 in univariable analyses and clinically relevant covariates were included in the multivariable models. Subgroup analyses were prespecified according to diabetes status (T2DM vs. non-T2DM) and baseline HSI categories (35–44, 45–54, ≥55).

All statistical analyses were conducted using complete-case data in IBM SPSS Statistics version 21. Patients with missing one-year outcome data were excluded from the corresponding analyses. A post hoc power estimation was conducted to assess the detectable effect size given the final sample size and observed outcome rate, using G*Power version 3.1.9.7. Assuming a two-sided α = 0.05, a sample size of 177, and an outcome rate of 33% for HSI normalization in our cohort, we estimated that the analysis had over 80% power to detect an odds ratio of 2.0 or greater in a logistic regression model. A two-tailed *p*-value < 0.05 was considered statistically significant.

## 3. Results

### 3.1. Study Population and Baseline Characteristics

A total of 198 adult patients who were enrolled in the Asan Bariatric and Metabolic Surgery Registry between January 2019 and April 2024 were initially screened for inclusion. Of these, five patients who developed complications were excluded at the outset of the inclusion process (wound complication, n = 1; incisional hernia, n = 3; diabetic ketoacidosis, n = 1). An additional 16 patients were subsequently excluded for the following reasons: surgery canceled before admission (n = 1); underwent Roux-en-Y gastric bypass (n = 1); no follow-up at all after surgery (n = 3), follow-up at 1 month only, followed by loss to follow-up (n = 7); follow-up at 3 months only (n = 2); and the postoperative use of anti-obesity medications (n = 2). As a result, 177 patients with complete two-week and 12-month data were included in the final analysis (Supplementary Figure S1).

The patients’ baseline characteristics are summarized in Table 1. The mean age of the cohort was 37.0 ± 9.4 years, and 41.2% were male. The mean baseline body weight and BMI were 113.2 ± 23.7 kg and 39.9 ± 6.6 kg/m^2^, respectively. The mean HSI at baseline was 51.5 ± 7.9. Among the participants, 79 (44.6%) had T2DM, 141 (79.7%) had hypertension, 107 (60.5%) had dyslipidemia, and 110 (62.1%) had fatty liver.

When stratified by diabetes status, patients with T2DM showed a significantly higher prevalence of dyslipidemia (71.8% vs. 51.5%, *p* = 0.006) and fatty liver (73.1% vs. 53.5%, *p* = 0.008) compared with those without T2DM. Their fasting plasma glucose and HbA1c levels were markedly higher (both *p* < 0.001), as were TG and LDL-C levels (*p* = 0.013 and *p* = 0.002, respectively), whereas HDL-C levels were lower (*p* = 0.002) in patients with T2DM. Other baseline parameters, including age, sex distribution, body weight, BMI, blood pressure, and liver/kidney function tests, were not significantly different between groups.

### 3.2. Longitudinal Changes in Metabolic Parameters

Changes in clinical and biochemical parameters during the one-year follow-up period are summarized in Table 2. Significant reductions in body weight and BMI were observed at 3, 6, and 12 months postoperatively (all *p* < 0.001). Glycemic parameters, including fasting glucose and HbA1c, also improved consistently at all follow-up points (all *p* < 0.001). TG levels were significantly reduced (*p* < 0.001), whereas TC and LDL-C showed no significant change. In contrast, HDL-C increased significantly at both 6 and 12 months (*p* < 0.001). Both AST and ALT levels decreased postoperatively, with ALT showing a more marked reduction. AST levels did not change at 3 months but significantly declined from 6 months onward (*p* < 0.001). The HSI improved significantly at all follow-up time points, decreasing from 51.5 ± 7.9 at baseline to 33.2 ± 5.8 at one-year (*p* < 0.001). In contrast, the FIB-4 index remained unchanged throughout the follow-up, with no significant differences compared with baseline at 3, 6, and 12 months.

### 3.3. Predictive Performance of Early Postoperative Weight Loss for One-Year Outcomes

**Figure 1:**
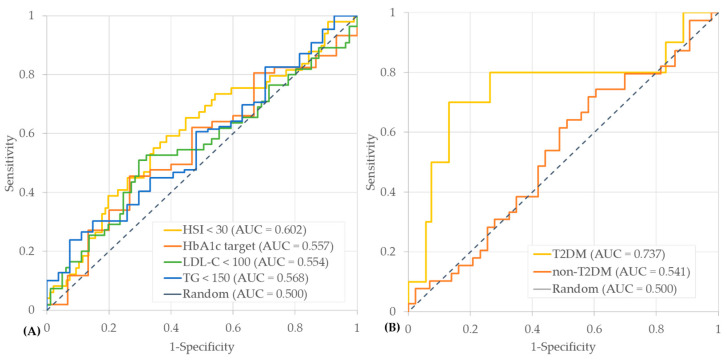
Receiver operating characteristic curves (ROCs) for early postoperative weight loss in predicting one-year metabolic outcomes. (**A**) Predictive performance of %TWL_2W for achieving target levels of HSI < 30, glycemic control (HbA1c < 6.5% for DM or <5.7% for non-DM), LDL-C < 100 mg/dL, and TG < 150 mg/dL. (**B**) Subgroup analysis of ROC curves for predicting HSI < 30 according to diabetes status.

**Figure 2:**
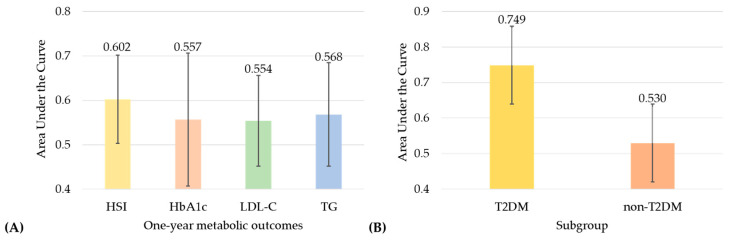
Bar plot of area under the receiver operating characteristic curve (AUC) for early postoperative weight loss in predicting one-year metabolic outcomes. (**A**) AUCs for four metabolic outcomes: HSI < 30, HbA1c target, LDL-C < 100 mg/dL, and TG < 150 mg/dL. Error bars represent 95% confidence intervals (CIs). (**B**) Subgroup comparison of AUC for HSI < 30 according to diabetes status (T2DM vs. non-T2DM). Error bars represent 95% CIs.

As shown in Figure 1A, early postoperative weight loss at 2 weeks demonstrated modest discriminatory ability in predicting one-year metabolic improvements. Of the outcomes assessed, the AUC was the highest for the HSI (HSI < 30, AUC = 0.602; 95% CI: 0.503–0.702; *p* = 0.044), followed by TG (TG < 150 mg/dL, AUC = 0.568; 95% CI: 0.452–0.685; *p* = 0.059), LDL-C (LDL-C < 100 mg/dL, AUC = 0.554; CI: 0.452–0.656; *p* = 0.284), and HbA1c (HbA1c < 6.5% for patients with DM or <5.7% for those without DM, AUC = 0.557; 95% CI: 0.407–0.706; *p* = 0.480) (Figure 2A). Optimal cut-offs for postoperative 2-week weight loss were identified using the Youden Index: HSI, 7.9%; HbA1c, 7.7%; LDL-C, 8.1%; TG, 9.4% (Supplementary Table S1). To further characterize these cut-offs, Supplementary Table S2 compares baseline and one-year outcomes between patients above and below the 7.9% threshold, which corresponded to the optimal cut-off derived for HSI, the outcome most significantly predicted by early weight loss. Patients with higher early weight loss (≥7.9%) were significantly younger at baseline, while the other clinical characteristics were comparable. At one-year, the high-early-weight-loss group showed greater improvement in the HSI (45.0% vs. 25.9%, *p* = 0.017) and a borderline trend toward achieving LDL-C target (50.0% vs. 33.3%, *p* = 0.050). In contrast, no significant group differences were observed for HbA1c or TG outcomes.

### 3.4. Association Between Early Weight Loss and One-Year Metabolic Improvements

Univariate logistic regression showed that early weight loss at or above the %TWL_2W cut-off was significantly associated with an improvement in the HSI (OR = 2.34; 95% CI: 1.16–4.73; *p* = 0.018) and LDL-C (OR = 2.46; 95% CI: 1.21–5.02; *p* = 0.013) (Table 3). No significant associations were observed for HbA1c or TG outcomes. Furthermore, to examine whether HSI, the one-year outcome most strongly associated with early two-week weight loss, showed consistent associations with improvements in other metabolic outcomes, we compared clinical parameters at one-year between individuals above and below the one-year HSI cut-off value of 30 (Supplementary Table S3). At one year, patients with HSI < 30 demonstrated significantly greater weight loss compared with those with an HSI ≥ 30 (one-year %TWL: 30.38 ± 7.64 vs. 22.40 ± 8.08, *p* < 0.001). In addition, the proportion of patients who achieved HbA1c improvement (97.4% vs. 82.3%, *p* = 0.020) and TG reduction (91.5% vs. 74.2%, *p* = 0.016) was significantly higher in the one-year HSI < 30 group. No significant differences were observed between groups in LDL-C improvement (48.9% vs. 36.0%, *p* = 0.142). These findings indicate that a favorable one-year HSI status was accompanied by greater weight loss and consistent improvements in selected metabolic parameters.

### 3.5. Subgroup Analyses by Hepatic Steatosis Severity and Diabetes Status

To assess whether baseline steatosis severity modified the predictive value of early weight loss, the patients were stratified by values of 35–44, 45–54, and ≥55 (Table 4). %TWL_2W ≥ 7.9% was significantly associated with achieving HSI < 30 at one-year only in the 45–54 group (OR = 3.56; 95% CI: 1.26–10.04; *p* = 0.017). No significant associations were observed in the other groups. Notably, only three patients with a baseline HSI ≥ 55 achieved HSI < 30 at one-year, suggesting markedly limited reversibility of hepatic steatosis in this subgroup.

In an exploratory analysis using a ≥35% relative reduction in HSI, greater early weight loss was associated with higher odds of liver fat improvement in patients with moderate and severe baseline steatosis (HSI 45–54: OR 3.83; HSI ≥ 55: OR 5.83; 95% CIs in Supplementary Table S4). In contrast, findings were not significant in the mild stratum. These results suggest that early postoperative weight loss relates to relative steatosis improvement even when full normalization is infrequent in advanced disease.

A subgroup analysis based on baseline T2DM status was performed to determine whether the effect of early postoperative weight loss on hepatic steatosis improvement (defined as HSI < 30 at one-year) differed according to the presence of diabetes. Figure 1B and Figure 2B show that %TWL_2W had greater predictive ability for HSI < 30 in patients with T2DM (AUC = 0.737; 95% CI: 0.548–0.950; *p* = 0.013) than in non-T2DM patients (AUC = 0.541; 95% CI: 0.404–0.656; *p* = 0.639). The difference between the two independent ROC curves approached statistical significance (∆AUC = 0.196; 95% CI: −0.004–0.442; *p* = 0.055; by Hanley and McNeil’s method). From a clinical perspective, net reclassification analysis in the T2DM subgroup demonstrated clear utility: among patients who achieved HSI < 30 at one-year, 80% were correctly classified by the model as having a high probability of HSI normalization, whereas among those who did not achieve HSI < 30, 72% were correctly classified as having a low probability of HSI normalization. As shown in Table 5, using a cut-off of 9.1% (Youden Index), early weight loss was significantly associated with hepatic improvement in patients with T2DM (OR = 13.42; 95% CI, 2.86–63.02; *p* = 0.001), whereas no significant association was observed in patients without T2DM (OR = 1.11; 95% CI, 0.42–2.95; *p* = 0.839). After adjusting for age, sex, baseline HbA1c, and BMI, this association remained significant in T2DM patients (adjusted OR = 9.12; 95% CI, 1.39–59.82; *p* = 0.021) but not in non-T2DM patients (adjusted OR = 0.70; 95% CI, 0.18–2.77; *p* = 0.611).

## 4. Discussion

This single-center retrospective cohort study evaluated 177 patients who underwent LSG between January 2019 and April 2024. The primary finding was that %TWL_2W emerged as the strongest early predictor of hepatic improvement at one-year. Specifically, a two-week weight loss of 7.9% most accurately predicted HSI normalization to <30, conferring an odds ratio of 2.34 compared with smaller early losses. Although ALT and AST showed wide variability at the 2-week time point, likely reflecting transient postoperative fluctuations, both enzymes demonstrated consistent and significant reductions thereafter, indicating sustained improvement in liver function. In contrast, the FIB-4 index did not show significant longitudinal changes in our dataset, and it exhibited a different trajectory compared with the HSI. While the HSI primarily reflects hepatic steatosis, FIB-4 is an estimator of hepatic fibrosis. In our cohort, the mean baseline HSI was elevated (51.50 ± 7.85), consistent with substantial steatosis, while the baseline FIB-4 index was relatively low (0.79 ± 0.55), suggesting limited fibrosis. This discrepancy likely explains why FIB-4 remained unchanged during postoperative follow-up, indicating that the patients had considerable steatosis but minimal fibrosis at baseline.

A dose–response relationship was also evident: patients with moderate steatosis (HSI 45–54) who achieved ≥ 7.9% early weight loss were three times more likely to normalize their HSI, whereas only a small minority with severe steatosis (HSI ≥ 55) attained full resolution. Predictive capacity was particularly pronounced in the T2DM subgroup, where the AUC rose to 0.737 and ≥9.1% early weight loss conferred an adjusted OR of 9.12. Collectively, these findings suggest that a simple two-week weight-loss milestone can help anticipate one-year hepatic outcomes after SG, particularly in patients with T2DM or moderate baseline steatosis. However, given the modest overall discrimination (HSI AUC 0.602; Youden 0.207), any single two-week cut-off has limited standalone utility. Accordingly, we report 7.9% as a provisional reference point rather than a prescriptive decision threshold, with the strongest performance observed in those prespecified contexts.

The present data indicate that losing roughly 8% of baseline weight within the first two postoperative weeks can serve as a pragmatic early marker for risk stratification after SG. Patients who do not attain this threshold may benefit from intensified dietary counseling provided at shorter intervals, coupled with the timely initiation—or dose escalation—of adjunct pharmacotherapies such as GLP-1 receptor agonists or sodium-glucose co-transporter 2 inhibitors. Embedding these measures within a predefined surveillance framework that incorporates follow-up liver imaging at three- to six-month intervals could help clinicians detect—and address—suboptimal hepatic responses before fibrosis progresses.

Patients presenting with severe steatosis (HSI ≥ 55) merit particular attention. In this stratum, complete normalization (HSI < 30) was uncommon, indicating limited absolute reversibility despite comparable relative weight loss. These outcome patterns are biologically plausible. Advanced steatosis is characterized by impaired mitochondrial function, diminished β-oxidative capacity, and progressive fibrotic remodeling, all of which restrict metabolic flexibility and the ability to clear intra-hepatic fat despite similar weight loss percentages [31,32,33]. This pathophysiological ceiling is consistent with our finding that only 3 of 49 individuals in the highest HSI stratum reached complete normalization at 12 months. Nevertheless, an exploratory analysis using a ≥35% relative reduction in the HSI demonstrated that greater early weight loss was associated with higher odds of liver fat improvement in both moderate and severe baseline steatosis. Thus, although complete reversal is infrequent in advanced disease, clinically meaningful relative improvement remains attainable.

Beyond hepatic outcomes, early weight loss also showed relevance for lipid targets. Patients with greater early postoperative weight loss were more likely to achieve LDL-C < 100 mg/dL at one year, despite a lack of significant group-level reduction in mean LDL-C. This observation is consistent with prior studies describing heterogeneous lipid responses after sleeve gastrectomy and suggests that very early weight trajectories may influence the probability of reaching lipid goals in individual patients [34]. Taken together, these findings highlight the broader metabolic relevance of early weight loss, extending its prognostic value beyond hepatic outcomes.

Multiple groups have shown that the pace of weight reduction immediately after bariatric surgery forecasts longer-term outcomes. After both SG and RYGB, early loss at 1 or 3 months explains up to 30–50% of the variance in maximal weight loss at 1–5 years and helps flag “slow losers” who may benefit from intensified follow-up [14,35,36,37]. Our finding that a two-week total weight loss of 8–9% predicts hepatic benefit therefore extends this concept from global weight trajectories to organ-specific metabolic endpoints.

Meta-analyses consistently demonstrate marked resolution of NAFLD features after metabolic surgery, with RYGB achieving long-term reductions in liver fat, inflammation, and fibrosis, including steatosis resolution in up to 95% of cases, while SG provides comparable hepatic benefits, significantly improving steatosis and fibrosis in approximately 60% of patients [38,39,40,41]. Importantly, most pooled analyses report aggregate mean changes without stratification by baseline steatosis severity or diabetes status. It remains unclear whether patients with advanced disease or dysglycemia experience comparable benefit, a critical research gap that our stratified HSI data help to fill.

During the first two postoperative weeks, patients typically consume an energy-restricted liquid diet (≈400–600 kcal per day) and experience an abrupt drop in insulin, creating an acute negative energy balance. In individuals with T2DM, this state appears to mobilize intrahepatic TG more rapidly than in their euglycemic peers, likely because higher basal hepatic fat content and insulin resistance amplify the gradient for lipid efflux [42,43,44]. Under hypocaloric conditions, hepatic de novo lipogenesis is sharply suppressed, while very-low-density lipoprotein export accelerates, yielding ≈30% reductions in liver fat within 7 days in T2DM cohorts with normalization of fasting glucose and de novo lipogenesis markers. In contrast, non-diabetic participants exhibit slower, smaller shifts [45,46]. In our dataset, the hepatic response is reflected indirectly in prediction metrics: the AUC for early weight loss increased from 0.602 in the full cohort to 0.737 in the T2DM subgroup, suggesting that rapid postoperative weight loss confers a large hepatic benefit, particularly in patients with diabetes. Taken together, two-week weight loss functions both as a surrogate of surgical efficacy and as a window into individual metabolic flexibility, especially in the context of diabetes.

SG also elicits large, early rises in gut-derived hormones, such as glucagon-like peptide 1 (GLP-1), PYY, and FGF19, together with a two- to three-fold increase in circulating conjugated bile acids and a shift toward bile-tolerant microbial taxa [47]. GLP-1 and FGF19 enhance hepatic β-oxidation and down-regulate SREBP-1c, whereas secondary bile acids activate FXR and TGR, further curbing lipogenesis [48,49,50,51]. These convergent signals magnify the lipid-lowering impact of caloric restriction and may explain why relatively modest early weight loss translates to a pronounced hepatic dividend [52].

This study possesses several distinctive strengths. Conducted at a high-volume Korean center, it represents one of the largest Asian series to provide uninterrupted 12-month biochemical surveillance after sleeve gastrectomy, thereby complementing the predominantly Western evidence base. By translating an easily measurable two-week weight loss milestone into a clinically actionable predictor of hepatic outcome, this study offers a practical tool for early postoperative management. Stratified analyses by baseline steatosis severity and diabetes status further illuminate the patient subgroups most likely to benefit from rapid early weight reduction. Finally, this study’s use of prospectively captured electronic medical record data and multivariable modeling that adjusted for age, sex, HbA1c, and BMI strengthens the credibility of the associations reported.

Several caveats warrant consideration. First, the retrospective, single-center design and restriction to patients undergoing sleeve gastrectomy may limit generalizability and introduce selection bias. External validation in independent cohorts has not yet been performed. Second, hepatic steatosis was assessed using the HSI, a pragmatic and widely used surrogate in large retrospective cohorts. However, the HSI is less precise than imaging or histologic evaluation and is not currently recommended as a standalone diagnostic or monitoring tool by major international guidelines. Imaging-based quantification or liver biopsy was not routinely available in our cohort, as the patients were enrolled primarily for the management of obesity or T2DM and not for suspected liver disease. Furthermore, liver imaging is not uniformly performed preoperatively in bariatric practice in Korea due to limited reimbursement coverage under the national health insurance system. As a result, comprehensive imaging or biopsy data were not consistently available for all participants, limiting our ability to validate steatosis resolution through gold-standard methods. Third, postoperative lifestyle and treatment adjustments such as dietary intake, physical activity, alcohol use, and changes in concomitant medications were not systematically captured, and information on the patients’ use of vitamin and mineral supplements was also limited, leaving room for residual confounding. Lastly, subgroup analyses should be interpreted with caution: among patients with severe baseline steatosis (HSI ≥ 55), only three individuals achieved HSI normalization at one year, yielding wide confidence intervals and unstable regression estimates. This likely reflects both the biological difficulty of reversing advanced steatosis and the statistical limitation imposed by low event counts.

In conclusion, weight loss of approximately 7.9–9.1% in the first two postoperative weeks was independently associated with one-year hepatic improvement, with the strongest associations observed in patients with T2DM. Given the modest overall discrimination (HSI AUC 0.602), this early metric should be interpreted as an associative risk indicator rather than a definitive predictor. It may assist with early risk stratification and individualized postoperative management after SG, helping clinicians consider earlier supportive measures in patients at risk of suboptimal hepatic response. Given that this 2-week cut-off is a provisional reference, prospective external validation using imaging-based endpoints remains warranted to establish its clinical applicability.

## Figures and Tables

**Table 1:** Baseline demographic, clinical and biochemical characteristics of the study participants in the overall cohort and stratified by presence of type 2 diabetes.

	Overall Cohort	T2DM	Non-T2DM	*p*-Value
Age (years)	37.01 ± 9.38	38.03 ± 10.30	36.21 ± 8.48	0.212
Male	73 (41.2)	38 (48.7)	35 (35.4)	0.073
Comorbidities				
T2DM	79 (44.6)			
Hypertension	141 (79.7)	66 (84.6)	75 (75.8)	0.146
Dyslipidemia	107 (60.5)	56 (71.8)	51 (51.5)	0.006
Fatty liver	110 (62.1)	57 (73.1)	53 (53.5)	0.008
Clinical parameters				
Body weight (kg)	113.23 ± 23.71	113.71 ± 23.39	112.86 ± 24.07	0.814
BMI (kg/m^2^)	39.91 ± 6.61	39.60 ± 6.52	40.17 ± 6.70	0.565
SBP (mmHg)	145.87 ± 16.55	146.72 ± 17.01	145.20 ± 16.23	0.549
DBP (mmHg)	88.87 ± 11.81	88.03 ± 11.16	89.54 ± 12.33	0.394
Biological parameters				
FPG (mg/dL)	119.15 ± 38.40	139.21 ± 48.48	103.35 ± 15.14	<0.001
HbA1c (%)	6.37 ± 1.34	7.34 ± 1.45	5.59 ± 0.42	<0.001
C-peptide (ng/mL)	4.28 ± 2.26	4.70 ± 2.86	3.95 ± 1.54	0.043
TC (mg/dL)	180.19 ± 38.62	169.37 ± 41.66	188.70 ± 33.88	0.001
TG (mg/dL)	176.70 ± 108.63	199.50 ± 111.31	158.36 ± 103.37	0.013
HDL-C (mg/dL)	44.61 ± 9.64	42.22 ± 8.47	46.54 ± 10.12	0.002
LDL-C (mg/dL)	113.92 ± 35.18	104.58 ± 37.35	121.43 ± 31.56	0.002
AST (U/L)	36.11 ± 26.70	41.97 ± 33.01	31.50 ± 19.36	0.014
ALT (U/L)	44.23 ± 37.22	50.68 ± 43.77	39.14 ± 30.39	0.050
Creatinine (mg/dL)	0.86 ± 0.73	0.96 ± 1.09	0.79 ± 0.16	0.179
eGFR (mL/min/1.73 m^2^)	104.75 ± 18.74	104.18 ± 23.24	105.20 ± 14.35	0.734
HSI	51.50 ± 7.86	52.24 ± 7.22	50.92 ± 8.32	0.263
FIB-4	0.79 ± 0.55	0.92 ± 0.68	0.69 ± 0.40	0.008

Note: values are expressed as means ± standard deviations (SDs) or numbers (%), unless otherwise indicated. *p*-values represent between-group differences (T2DM vs. non-T2DM). Abbreviation: T2DM, type 2 diabetes mellitus; BMI, body mass index; SBP, systolic blood pressure, DBP, diastolic blood pressure; FPG, fasting plasma glucose; HbA1c, glycated hemoglobin; TC, total cholesterol; TG, triglyceride; HDL-C. high-density lipoprotein cholesterol; LDL-C, low-density lipoprotein cholesterol; AST, aspartate aminotransferase; ALT, alanine aminotransferase; eGFR, estimated glomerular filtration rate; HSI, hepatic steatosis index; FIB-4, fibrosis-4 index.

**Table 2:** Changes in clinical and biochemical parameters before and after sleeve gastrectomy.

	Pre-OP	Post-OP 2 W	Post-OP 3 M	*p*-Value	Post-OP 6 M	*p*-Value	Post-OP 1 Year	*p*-Value
Body weight	113.33 ± 23.70	104.28 ± 23.67	93.75 ± 20.35	<0.001 **	88.17 ± 20.13	<0.001 **	84.85 ± 19.31	<0.001 **
BMI	39.91 ± 6.62	30.92 ± 6.18	27.83 ± 5.31	<0.001 **	26.11 ± 5.27	<0.001 **	25.15 ± 5.03	<0.001 **
%TWL_2W		7.90 ± 6.62						
FBS	119.15 ± 38.3		102.41 ± 22.77	<0.001 **	97.53 ± 16.26	<0.001 **	98.67 ± 23.18	<0.001 **
HbA1c	6.37 ± 1.34		5.61 ± 0.86	<0.001 **	5.54 ± 0.74	<0.001 **	5.59 ± 0.85	<0.001 **
TC	180.19 ± 38.61		179.60 ± 35.19	0.724	183.67 ± 39.11	0.391	181.38 ± 33.67	0.990
TG	176.70 ± 108.62		123.37 ± 54.31	<0.001 **	113.66 ± 59.10	<0.001 **	109.45 ± 53.34	<0.001 **
HDL-C	44.61 ± 9.63		48.75 ± 33.62	0.167	51.75 ± 10.56	<0.001 **	57.67 ± 13.11	<0.001 **
LDL-C	113.92 ± 35.17		116.31 ± 30.74	0.199	115.46 ± 33.60	0.447	107.38 ± 29.53	0.143
AST	36.11 ± 26.69		29.02 ± 65.53	0.279	20.93 ± 7.11	<0.001 **	20.65 ± 6.75	<0.001 **
ALT	44.23 ± 37.22		26.58 ± 73.02	0.009 *	15.41 ± 9.65	<0.001 **	15.91 ± 10.53	<0.001 **
HSI	51.50 ± 7.85		36.22 ± 6.86	<0.001 **	33.84 ± 6.44	<0.001 **	33.15 ± 5.82	<0.001 **
FIB-4	0.79 ± 0.55		0.80 ± 0.47	0.761	0.83 ± 0.46	0.549	0.88 ± 0.45	0.064

Abbreviations: OP, operation; 2 W, 2 weeks; 3 M, 3 months; 6 M, 6 months; %TWL_2W, percentage of total weight loss at 2 weeks. * *p* < 0.05 and ** *p* < 0.001 by paired *t*-test compared with preoperative values at 3, 6, and 12 months.

**Table 3:** Association between early weight loss and improvement in one-year metabolic outcomes.

Outcome	OR (95% CI)	*p*-Value
HSI	2.34 (1.16–4.73)	0.0176 *
LDL-C	2.46 (1.21–5.02)	0.0131 *
HbA1c	2.31 (0.69–7.73)	0.1749
TG	3.92 (0.87–17.66)	0.0757

Note: *p*-values are from two-sided Wald tests of OR = 1. * *p* < 0.05.

**Table 4:** Association between early postoperative weight loss and one-year hepatic steatosis improvement, stratified by baseline HSI group.

Baseline HSI	OR (95% CI)	*p*-Value
35–44	1.52 (0.29–8.03)	0.6254
45–54	3.56 (1.26–10.04)	0.0167 *
≥55	0.94 (0.08–11.15)	0.9596

Note: *p*-values are from two-sided Wald tests of OR = 1. * *p* < 0.05.

**Table 5:** Univariate and multivariate logistic regression analyses for predicting one-year hepatic steatosis improvement (HSI < 30) according to early postoperative weight loss (≥9.1%), stratified by diabetes status.

Group	OR (95% CI)	*p*-Value	Model
T2DM	13.42 (2.86–63.02)	0.0010 **	unadjusted
	9.12 (1.39–59.82)	0.0212 *	adjusted
Non-T2DM	1.11 (0.42–2.95)	0.8386	unadjusted
	0.70 (0.18–2.77)	0.6108	adjusted

Note: *p* for interaction between early weight loss (≥9.1%) and diabetes status was 0.002 in the unadjusted model and 0.005 in the adjusted model. * *p* < 0.05 and ** *p* < 0.001.

## Data Availability

Datasets used and/or analyzed during the current study are available from the corresponding author on reasonable request.

## Supplementary Materials

The following supporting information can be downloaded at: https://www.mdpi.com/article/10.3390/jcm14207284/s1, Figure S1: Flow diagram of study cohort selection; Table S1: Optimal cut-off values of 2-week total weight loss for predicting one-year metabolic targets based on receiver operating characteristics (ROC) analysis; Table S2: Baseline characteristics and one-year outcomes according to early postoperative weight loss (%TWL_2W ≥ 7.9% vs. <7.9%); Table S3: Comparison of metabolic outcomes at one-year in patients with HSI < 30 versus HSI ≥ 30; Table S4: Association between early postoperative weight loss and one-year ≥ 35% reduction in HSI, stratified by baseline HSI group.
