# Supplementary material for: Association of Rapid Early Weight Loss with One-Year Hepatic Steatosis Improvement After Sleeve Gastrectomy: A Retrospective Cohort Study
Source: J Clin Med. 2025 Oct 15;14(20):7284. doi: 10.3390/jcm14207284 (PMC12565118; doi:10.3390/jcm14207284)
Supplement: Supplementary file 1 [file jcm-14-07284-s001.zip › jcm-3841425-supplementary.pdf]

Journal of Clinical Medicine

MDPI

Supplementary Data.

Supplementary Figure 1. Flow diagram of study cohort selection.

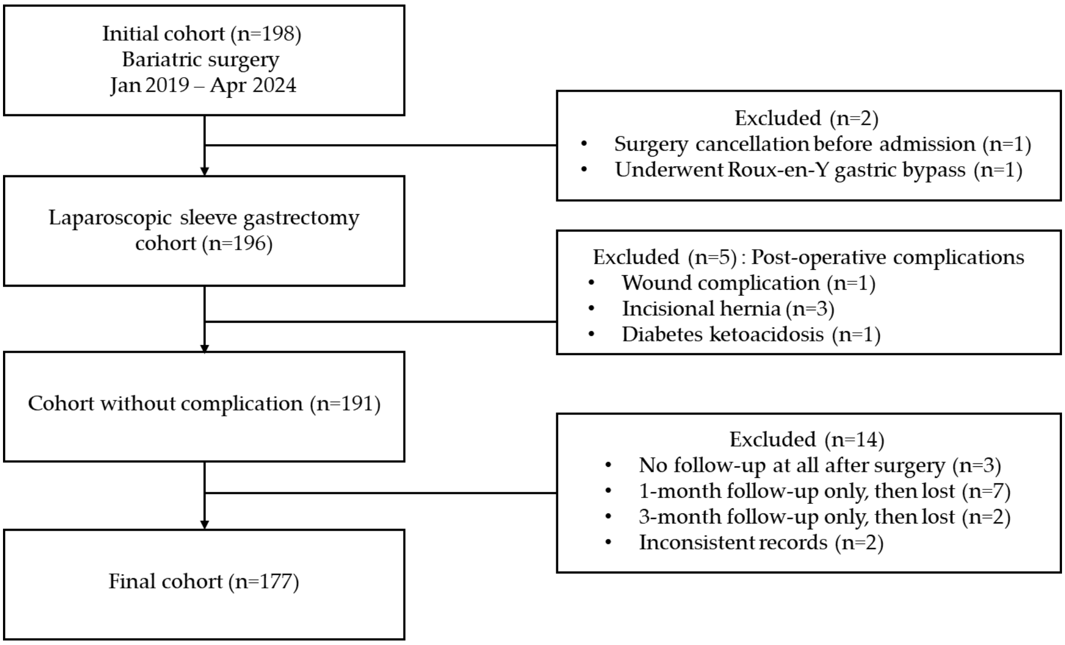

Supplementary Table 1. Optimal cut-off values of 2-week total weight loss for predicting one-year metabolic targets based on receiver operating characteristics (ROC) analysis.

Outcome target	AUC	Cut-off (%TWL_2W)	Sensitivity (%)	Specificity (%)	Youden Index
HbA1c	0.557	7.75	45.6	73.3	0.190
HSI	0.602	7.91	55.1	65.6	0.207
LDL-C	0.554	8.15	50.9	70.4	0.213
TG	0.568	9.45	23.9	92.6	0.164

Abbreviation: HbA1c, glycated hemoglobin; HSI, hepatic steatosis index; LDL-C, low density lipoprotein cholesterol; TG, triglyceride; AUC, area under the curve; %TWL_2W, percentage of total weight loss at 2 weeks.

Supplementary Table 2. Baseline characteristics and one-year outcomes according to early postoperative weight loss (%TWL_2W ≥ 7.9% vs < 7.9%).

	High%TWL_2W (≥ 7.9%)	Low%TWL_2W (< 7.9%)	*p*-value
Baseline			
Age (years)	34.72 ± 7.94	38.58 ± 9.32	0.007
Male	24 (33.3)	49 (46.7)	0.077
BMI (kg/m^2)	38.84 ± 5.76	40.66 ± 7.06	0.072
T2DM	28 (38.9)	50 (47.6)	0.250
Fatty liver	49 (68.1)	61 (58.1)	0.180
HbA1c (%)	6.20 ± 1.27	6.49 ± 1.38	0.151
HSI	50.44 ± 7.05	52.24 ± 8.32	0.135
FIB-4	0.71 ± 0.38	0.85 ± 0.64	0.072
LDL-C (mg/dL)	112.55 ± 31.40	114.88 ± 37.71	0.667
TG (mg/dL)	175.04 ± 107.63	177.85 ± 109.83	0.867

https://doi.org/10.3390/xxxxx

one-year target			
HbA1c	41 (91.1)	62 (84.9)	0.328
HSI	27 (45)	22 (25.9)	0.017
LDL-C	29 (50)	26 (33.3)	0.050
TG	49 (84.5)	60 (76.9)	0.274

Abbreviation: BMI, body mass index; T2DM, type 2 diabetes mellitus, FIB-4, fibrosis-4 index.

Notes: Values are presented as mean ± SD or number (%). P-values were calculated using independent t-test for continuous variables and chi-square tests for categorical variables. HbA1c target was defined as HbA1c < 6.5% in T2DM patients or < 5.7% in those without T2DM. LDL-C target was defined as LDL-C < 100mg/dL, TG target as TG < 150mg/dL, and HSI target as HSI < 30.

Abbreviation: BMI, body mass index.

Supplementary Table 3. Comparison of metabolic outcomes at one-year in patients with HSI < 30 versus HSI ≥ 30.

One-year target	HSI < 30	HSI ≥ 30	*p*-value
%TWL_1Y	30.38 ± 7.64	22.40 ± 8.08	0.328
HbA1c	38 (97.4)	65 (82.3)	0.017
LDL-C	23 (48.9)	32 (36.0)	0.050
TG	43 (91.5)	66 (74.2)	0.274

Supplementary Table 4. Association between early post-operative weight loss and one-year ≥ 35% reduction in HSI, stratified by baseline HSI group.

Baseline HSI	OR (95% CI)	*p*-value
35-44	1.37 (0.3-6.4)	0.6849
45-54	3.83 (1.38-10.69)	0.0102
≥ 55	5.83 (1.14-29.84)	0.0342
